# Wheat CBL-interacting protein kinase 25 negatively regulates salt tolerance in transgenic wheat

**DOI:** 10.1038/srep28884

**Published:** 2016-06-30

**Authors:** Xia Jin, Tao Sun, Xiatian Wang, Peipei Su, Jingfei Ma, Guangyuan He, Guangxiao Yang

**Affiliations:** 1 The Genetic Engineering International Cooperation Base of Chinese Ministry of Science and Technology, The Key Laboratory of Molecular Biophysics of Chinese Ministry of Education, College of Life Science and Technology, Huazhong University of Science & Technology, Wuhan 430074, China

## Abstract

CBL-interacting protein kinases are involved in plant responses to abiotic stresses, including salt stress. However, the negative regulating mechanism of this gene family in response to salinity is less reported. In this study, we evaluated the role of TaCIPK25 in regulating salt response in wheat. Under conditions of high salinity, *TaCIPK25* expression was markedly down-regulated in roots. Overexpression of *TaCIPK25* resulted in hypersensitivity to Na^+^ and superfluous accumulation of Na^+^ in transgenic wheat lines. *TaCIPK25* expression did not decline in transgenic wheat and remained at an even higher level than that in wild-type wheat controls under high-salinity treatment. Furthermore, transmembrane Na^+^/H^+^ exchange was impaired in the root cells of transgenic wheat. These results suggested that TaCIPK25 negatively regulated salt response in wheat. Additionally, yeast-one-hybrid, β-glucuronidase activity and DNA-protein-interaction-enzyme-linked-immunosorbent assays showed that the transcription factor TaWRKY9 bound W-box in the *TaCIPK25* promoter region. Quantitative real-time polymerase chain reaction assays showed concomitantly inverted expression patterns of *TaCIPK25* and *TaWRKY9* in wheat roots under salt treatment, ABA application and inhibition of endogenous ABA condition. Overall, based on our results, in a salt stress condition, the negative salt response in wheat involved TaCIPK25 with the expression regulated by TaWRKY9.

High soil salinization is one of the conditions that most restrict plant growth and has serious effects on the cultivation of cereal crops worldwide. High concentrations of sodium ions (Na^+^) negatively affect cellular metabolism and cause toxicity to plants. Therefore, emergency mechanisms must be initiated in plants to maintain low levels of Na^+^ under salt stress. The mechanism for Na^+^ tolerance depends on ion channels or carrier type transporters, particularly the Na^+^/H^+^ antiporters localized in either the tonoplast or plasma membrane^1^. AtNHX1 was first identified as a Na^+^/H^+^ antiporter in the vacuole membrane with five additional Arabidopsis AtNHXs characterized as homologs of AtNHX1 in subsequent studies^2^,^3^,^4^. With the overexpression of *AtNHX1*, salt tolerance in Arabidopsis increases significantly^2^, supported by T-DNA insertion of *nhx1* into Arabidopsis^5^. In recent years, according to many additional publications, the NHX channel has potassium-proton exchange activity in addition to Na^+^/H^+^ exchange in tonoplast vesicles^6^,^7^,^8^. Indeed, the role of vacuolar NHX channels in tolerance to salinity is apparently predominantly to maintain intracellular K^+^ homeostasis rather than to transport Na^+^ into a vacuole^9^.

For salt tolerance, the role of Na^+^/H^+^ antiporters in the plasma membrane has been demonstrated in Arabidopsis, soybean, wheat and rice^10^,^11^,^12^,^13^,^14^. The salt overly-sensitive (SOS) pathway related to calcineurin B-like protein (CBL)/CBL-interacting protein kinase (CIPK) network is well characterized as a representative Na^+^ efflux mechanism at the plasma membrane in Arabidopsis. To increase salt tolerance in plants, SOS3 (CBL4) protein interacts and recruits SOS2 (CIPK24) to the cytoplasmic membrane in which it activates the Na^+^/H^+^ (SOS1) antiporter by phosphorylation of the C-terminal auto-inhibitory domain^15^,^16^,^17^. Moreover, CBL/CIPK perceive cytosolic Ca^2+^ signals resulting from salt stress and have important roles in regulating salt stress response and ion homeostasis^18^,^19^,^20^,^21^,^22^,^23^.

In previous studies, Na^+^/H^+^ antiporters were activated haphazardly to determine the mechanism for the improved salt tolerance phenotype in transgenic plants with CBL/CIPK overexpressed^24^,^25^,^26^,^27^. However, the impaired salt tolerance of CBL/CIPK transgenic lines related to Na^+^ homeostasis was not reported. Abscisic acid (ABA)-dependent and -independent pathways, several transcription factors and promoter elements have also been identified in salt-stress signaling of plants^28^. However, the detailed regulation of *CIPK*s in these signaling pathways is less characterized. In a previous study, we isolated a wheat *CIPK*, i.e., *TaCIPK25* (homology to AtCIPK12, 18 and 19)^29^. In this study, based on our results, the overexpression of *TaCIPK25* impaired salt tolerance, which was mediated by a WRKY transcription factor in an ABA-dependent pathway under saline conditions.

## Results

### Expression patterns of *TaCIPK25* in response to salt and ABA application

**Figure 1:**
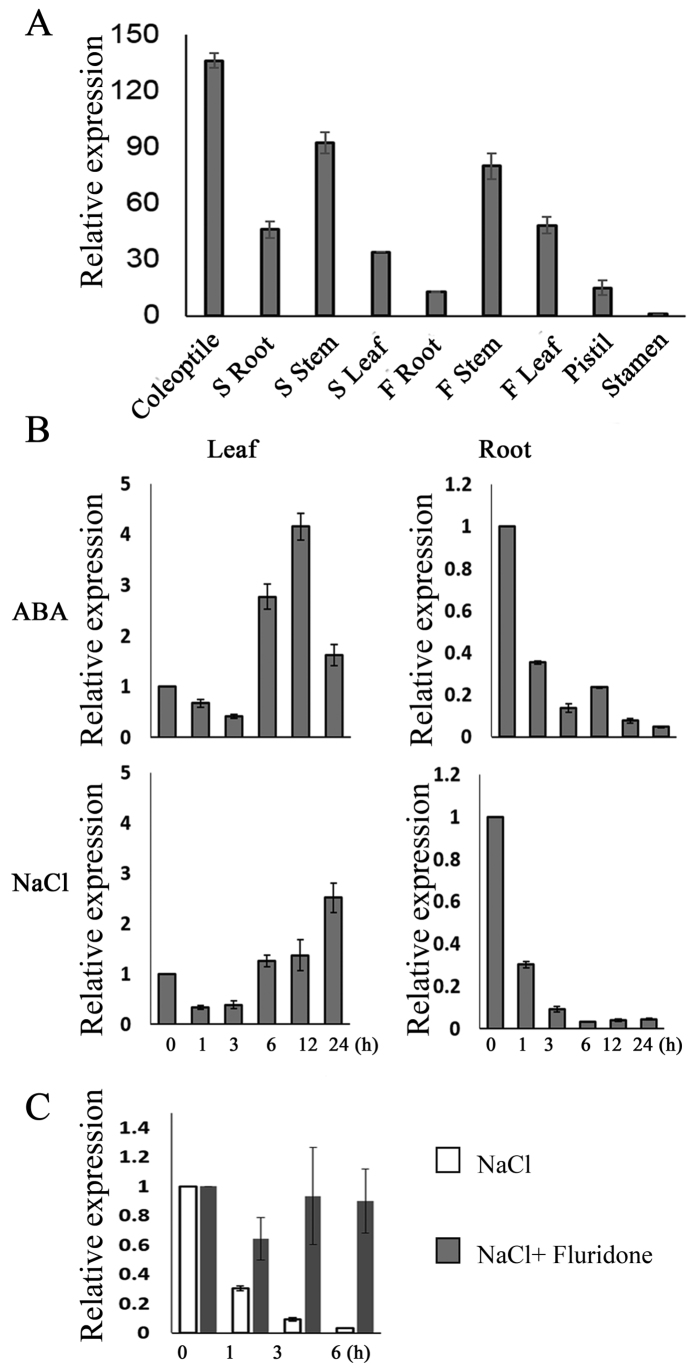
Expression patterns of *TaCIPK25* in wheat by qPCR. (**A**) gene transcript levels in wheat tissues. “S” and “F” represent seedlings and flowering stages, respectively. (**B**) gene expression analyses in roots and leaves responding to exogenous ABA (10 μM) and NaCl (200 mM). (**C**) comparative analyses of gene expression levels in wheat roots under NaCl (200 mM) and NaCl (200 mM) + fluridone (10 μM, an inhibitor of ABA biosynthesis). The Y-axis represents the relative expression levels compared with stamen (**A**) and controls (**B,C**). The transcript level in stamen relative to housekeeping gene is 0.00553. Three biological experiments were performed. Bars represent the mean ± SD (n = 3).

To analyze the expression patterns of *TaCIPK25*, the relative expression levels of *TaCIPK25* in different wheat tissues were detected using fluorescent quantitative analysis (qRT-PCR). *TaCIPK25* was expressed in all assayed tissues, including coleoptile, root, stem, leaf, pistil and stamens (Fig. 1A). To determine whether TaCIPK25 plays role in responses to abiotic stresses in wheat, the expression patterns of *TaCIPK25* in seedling roots and leaves were detected under ABA (100 μM) and NaCl (200 mM) treatments (Fig. 1B). The expression level of *TaCIPK25* in leaves initially decreased slightly, then was up-regulated at 6 h and increased to peak levels of expression under ABA and NaCl treatments. For gene expression in roots, expression of *TaCIPK25* decreased rapidly in 1 h and then decreased consistently to the lowest level of expression at 24 h in both NaCl and ABA treatments.

ABA is a well-known signal molecule in abiotic stress responses and regulates many stress-responsive genes^30^. To explore whether the expression of *TaCIPK25* in response to NaCl was related to the ABA-dependent pathway, we used fluridone to inhibit endogenous ABA biosynthesis. Under high salt treatment, the down-regulation of *TaCIPK25* expression was impaired in fluridone pretreated seedling roots compared with untreated seedling roots (Fig. 1C). Based on these results, TaCIPK25 down-regulated expressions induced by NaCl were involved in ABA signaling pathway.

### TaWRKY9 binding to the promoter region of *TaCIPK25*

**Figure 2:**
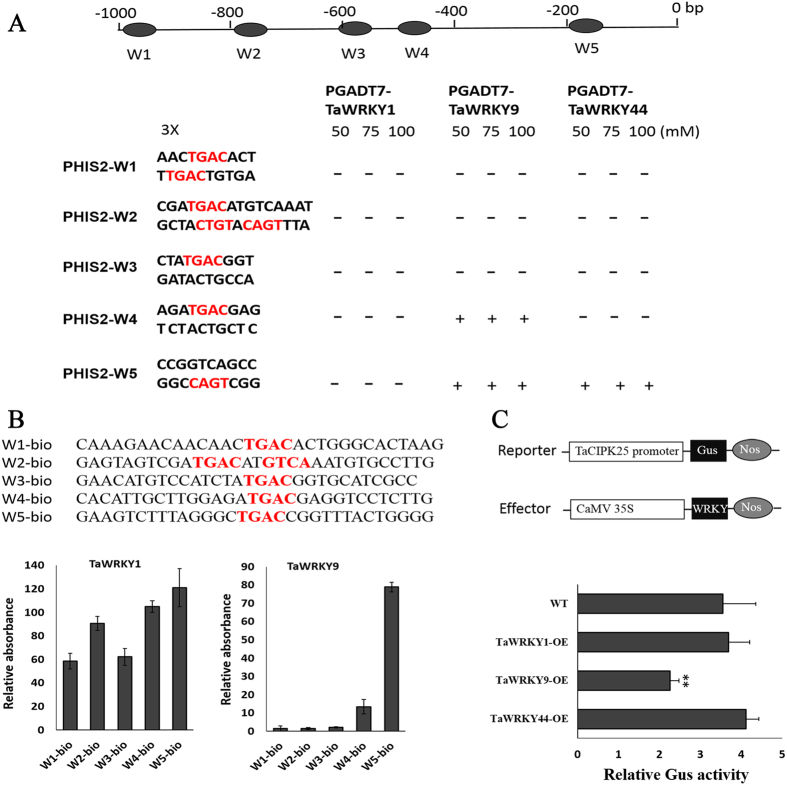
TaWRKYs interacted with the promoter of *TaCIPK25*. (**A**) interactions between W-boxes in promoter region of *TaCIPK25* and TaWRKYs (1, 9 and 44) by yeast one-hybrid assay. The up-panel represents the locations of five W-boxes (W1–W5) in 1000-bp promoter region of *TaCIPK25*. Three copies of each W-box elements were inserted into PHIS2.0 vector and *HIS3* gene was used as the reporter gene. “−” and “+” represent negative and positive interactions, respectively. **(B)**, DNA-binding capacity of TaWRKY1 and TaWRKY9 to the W-box probes. The W-boxes probes were biotinylated at 5′ ends (up-panel). The Y-axis represents the relative absorbance compared with negative control. **(C)**, transient expression of *TaCIPK25* promoter in tobacco leaves of *TaWRKY1, 9* and *44* transgenic lines. *TaWRKY*s were driven by *CaMV 35S* promoter as effector and *Gus* was driven by promoter of *TaCIPK25* as reporter (up-panel). Bars represent the mean ± SD (n = 3, **P < 0.01 by Student’s t-test). At least three biological experiments were performed.

*Cis*-elements play critical roles in the regulation of gene expression. Therefore, we isolated the 1,000-bp upstream sequence from the start codon of *TaCIPK25* and searched for putative elements using PLACE. We identified many putative *cis*-elements, including five putative WRKY transcription factor-binding sites (W1–W5; W-box core sequence, TGAC; Fig. 2A). WRKY transcription factors are both positive and negative regulators of ABA signaling and rapidly activate gene expression in plant defense processes^31^,^32^. In a previous study, we demonstrated that TaWRKYs were involved in improving stress tolerance in transgenic tobacco^33^,^34^; therefore, we selected three representative TaWRKY transcription factors (TaWRKY1, TaWRKY9 and TaWRKY44) to verify the interactions with promoters of *TaCIPK25*.

In a yeast one-hybrid (Y1H) assay, yeast cells transformed with three combinations (TaWRKY9-W4, TaWRKY9-W5, and TaWRKY44-W5) of TaWRKYs, and PHIS2-W-boxes grew well on the selective media (50–100 mM 3-AT; Fig. 2A and Supplementary Fig. 1), which was an indication that TaWRKY9 and TaWRKY44 physically interacted with the promoter of *TaCIPK25.* In our previous study using the Y1H method (positive control), TaWRKY44 interacted with the typical TTGACC element^34^, but this type of element was not found in the promoter region of *TaCIPK25*. No interaction between TaWRKY1 and W-boxes was detected in this analysis. Additionally, we used DPI-ELISA to further confirm these interactions; however, we did not analyze TaWRKY44 in this assay because we could not purify the recombinant protein effectively. As shown in Fig. 2B, TaWRKY1 gave a significant light absorption signal with the W1–W5 boxes, whereas TaWRKY9 had a strong signal only with the W5 element. Therefore, specific binding between TaWRKY9 and the W5 element was verified by both Y1H and DPI-ELISA methods. Notably, DPI-ELISA assay results were not completely consistent with those of the Y1H method, particularly for TaWRKY1. These differences might be related to the difference in protein purification method (purification in a prokaryotic expression system) with DPI-ELISA. We selected *Gus* gene as a reporter driven by the *TaCIPK25* promoter and transiently expressed the promoter-*Gus* construct in transgenic tobacco lines overexpressing *TaWRKY1, TaWRKY9*, and *TaWRKY44*. As shown in Fig. 2C, the relative Gus activities in the *TaWRKY1* and *TaWRKY44* transgenic lines had no significant differences compared with control lines. However, the relative Gus activity in *TaWRKY9* transgenic lines decreased compared with control lines, which again confirmed that TaWRKY9 acted on the promoter region of *TaCIPK25* by binding the W5 element and down-regulating the expression.

### Expression patterns of *TaWRKYs* in response to NaCl and ABA treatments

**Figure 3:**
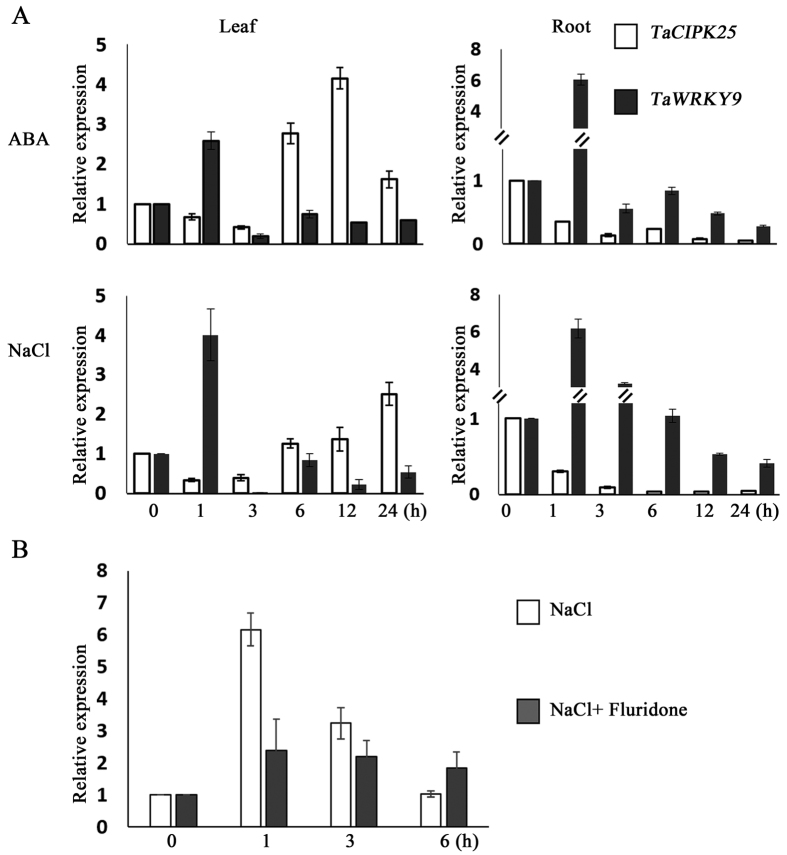
Comparative gene expression analyses of *TaCIPK25* and *TaWRKY9*. **(A)** gene expression analyses in wheat roots and leaves responding to exogenous ABA (10 μM) and NaCl (200 mM). **(B)** comparative gene expression analyses in wheat roots under NaCl (200 mM) + fluridone (10 μM). The Y-axis represents the relative expression levels compared with controls (0 h). Bars represent the mean ± SD (n = 3). Three biological experiments were performed.

To confirm the correlations of expression profiles between *TaCIPK25* and *TaWRKYs (TaWRKY1, 9* and *44*), the expression of *TaWRKY*s was also determined by qRT-PCR in wheat. Under ABA and NaCl treatments, *TaWRKY1* and *TaWRKY44* were up-regulated in leaves and down-regulated in roots (Supplementary Fig. 2). *TaWRKY9* was rapidly up-regulated and reached peak levels in 1 h in leaves and roots of wheat plants following ABA or NaCl treatment (Fig. 3A). Notably, the expression patterns of *TaWRKY9* were in contrast to those of *TaCIPK25* (Fig. 3B). Whereas the transcript levels of *TaWRKY9* rapidly accumulated in 1 h after treatment with NaCl and ABA, expression of *TaCIPK25* was down-regulated, particularly in roots. However, in saline conditions, expression patterns of *TaWRKY9* were inverted, when endogenous ABA was inhibited by fluridone (Fig. 3C). Based on these results, TaWRKY9 was a candidate transcription factor for the negative regulation of the expression of *TaCIPK25* by the ABA-dependent pathway under salt stress.

### Overexpression of *TaCIPK25* decreased salt tolerance and affected Na^+^ accumulation in Arabidopsis

**Figure 4:**
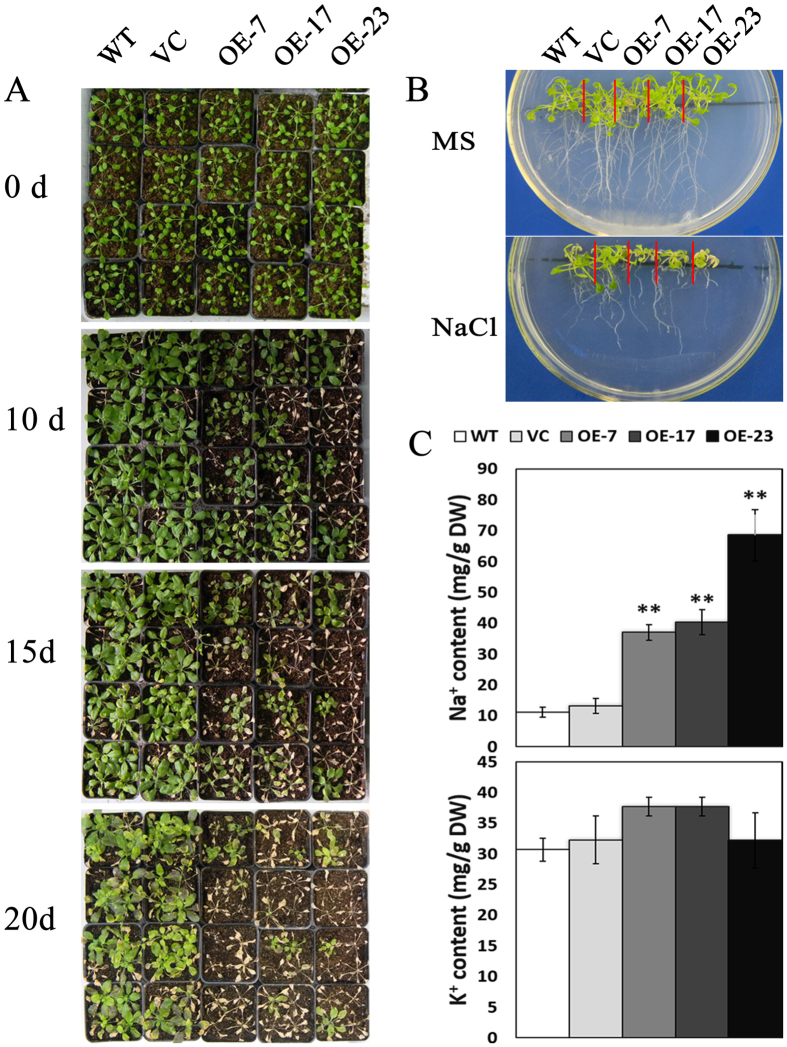
Salt tolerance assays in transgenic Arabidopsis lines over-expressed *TaCIPK25.* **(A)**, phenotypic assays in soil-grown Arabidopsis plants. Ten-day-old seedlings were irrigated with 200 mM NaCl at 5-day interval and the photos were taken at 10, 15 and 20 days after salt treatment. **(B)**, phenotypic assays of Arabidopsis seedlings in MS medium treated with 150 mM NaCl. C, Na^+^/K^+^ contents in soil grown Arabidopsis after 10 days salt treatment **(A)**. Bars represent the mean ± SD (n = 3, **P < 0.01 by Student’s t-test). Three biological experiments were performed.

Considering the low success rate of obtaining positive transgenic wheat for transformed genes, we first confirmed the salt tolerance performance in a model plant species. Transgenic Arabidopsis plants overexpressing *TaCIPK25* driven by CaMV 35S promoter were generated. Among these transgenic lines, OE7, OE17 and OE23 were randomly selected for further analysis. Overexpression of *TaCIPK25* in Arabidopsis was verified by RT-PCR; the three lines had different levels of *TaCIPK25* expression (Supplementary Fig. 3). For the salt tolerance assay, four-leaf stage, MS-germinated seedlings were transferred to MS medium or MS medium supplied with 150 mM NaCl. After 20 d, the growth phenotype of the transgenic lines was weaker than that of WT and vector control (VC) plants (Fig. 4B). For further analysis, 10-day-old seedlings of WT, VC and transgenic lines grown in soil were irrigated with 200 mM NaCl at 5-day intervals. As shown in Fig. 4A, the difference in growth status of transgenic lines and control plants began to appear 10 d after NaCl treatment. With an increase in time, leaf death was greater in transgenic lines than that in control plants. These results suggested that the tolerance of transgenic lines was impaired by overexpression of *TaCIPK25* in Arabidopsis.

Anti-oxidative systems are typically used to determine the salt tolerance of plants subjected to salt stress. In this study, the H_2_O_2_ content of transgenic lines was higher than those of controls (Supplementary Fig. 4). Expression of *TaCIPK25* was induced when wheat plants were treated with H_2_O_2_. To explore whether *TaCIPK25* participated in oxidative stress, the transgenic and control plants were treated with methyl viologen (causing oxidative damage); no obvious difference in the growth of plants between transgenic and control lines was observed within 20 d of treatment (Supplementary Fig. 5). These data suggested that the peroxides caused by salt stress were not responsible for the excessively withered leaves of transgenic Arabidopsis under a saline condition; thus, TaCIPK25 did not improve the tolerance of Arabidopsis to oxidative damage.

The phenotype with impaired salt tolerance of transgenic lines might be explained by the accumulation of Na^+^/K^+^. To determine whether this assumption was valid, the Na^+^/K^+^ content in leaves of the transgenic lines and control plants was measured following salt treatment. Based on the results, the accumulation of Na^+^ was higher in transgenic plants than that in controls, whereas the content of K^+^ was not significantly different (Fig. 4C). Therefore, the overexpression of *TaCIPK25* resulted in a greater accumulation of Na^+^ in transgenic Arabidopsis.

### Generation of transgenic wheat with overexpression of *TaCIPK25*

**Figure 5:**
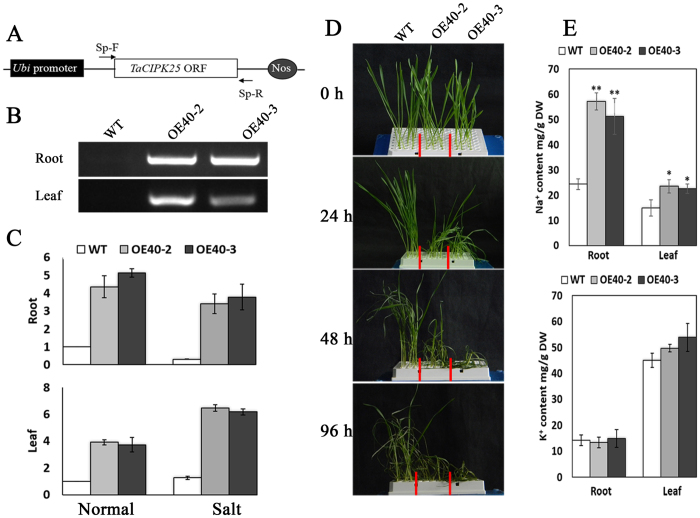
*TaCIPK25* was overexpressed in wheat. **(A)***TaCIPK25* was driven by *Ubi* promoter. “Sp-F” and “Sp-R” represent the specific primers to detect the gene expression from this construct. **(B)** PCR analyses in roots and leaves of transgenic lines with the specific primers. **(C)** expression analyses of *TaCIPK25* (total expression levels including endogenous and exogenous expressions of *TaCIPK25*) by qPCR. **(D)** salt tolerance assays in seedlings of wheat treated with 150 mM NaCl. (**E**) Na^+^/K^+^ contents in wheat roots and leaves after 24 h salt treatment. At least three biological experiments were performed. Bars represent the mean ± SD (n = 3, *P < 0.05, **P < 0.01 by Student’s t-test).

The ectopic expression of *TaCIPK25* in Arabidopsis provided a preliminary view of *TaCIPK25* function, whereas the actual role of TaCIPK25 depends on performance in wheat. Therefore, the CDS of *TaCIPK25* was constructed into an overexpression vector (OV-TaCIPK25) driven by the maize *ubiquitin* promoter. To detect transcripts of *TaCIPK25* derived from OV-TaCIPK25, specific primers were added to bilateral sites of the insertion gene (Fig. 5A). The OV-TaCIPK25 DNA was bombarded into wheat (Chinese spring cultivar) immature embryos to generate *TaCIPK25*-overexpressing transgenic wheat plants. Five positive transgenic T_0_ plants were identified by PCR with transgene-specific primers, and PCR products were sequenced to confirm the validity. When the transgenic lines were proliferated to T_2_ progenies, two stable transgenic lines, OE40-2 and OE40-3, were obtained with transcripts of *TaCIPK25* (derived from OV-TaCIPK25) detected in both lines by PCR assay. We found expression of *TaCIPK25* in root and leaf tissues (Fig. 5B). Based on qRT-PCR results, the *TaCIPK25* transgenic lines had much higher levels of *TaCIPK25* expression than that of the wild type under normal conditions (Fig. 5C). Notably, the transcript levels of *TaCIPK25* in transgenic lines did not decrease following treatment with 150 mM NaCl, which suggested that these two transgenic wheat lines could be used in further analyses.

### Overexpression of *TaCIPK25* impaired salt tolerance in wheat

To evaluate the effect of TaCIPK25 on salinity tolerance in wheat, wild type and transgenic lines were germinated and grown in MS liquid medium, and then 10-day-old seedlings were transferred to MS liquid medium containing 150 mM NaCl. Transgenic wheat plants withered on MS liquid medium containing 150 mM NaCl after 24 h treatment and were almost dead after 96 h, whereas WT plants grew normally (Fig. 5D). Based on these results, the overexpression of *TaCIPK25* significantly impaired salt tolerance in wheat. Twenty-four hours after salt treatment, the Na^+^/K^+^ content in roots and leaves of wild type and transgenic seedlings was measured. As shown in Fig. 5E, the accumulation of Na^+^ in the roots and leaves of the transgenic lines increased compared with that in those of wild-type controls (the K^+^ content was not significantly different). These results were similar to those observed in transgenic Arabidopsis (Fig. 4C); thus, the excess accumulation of Na^+^ was an indication of impaired salt tolerance in transgenic wheat.

### TaCIPK25 specific regulation of Na^+^ accumulation in wheat

**Figure 6:**
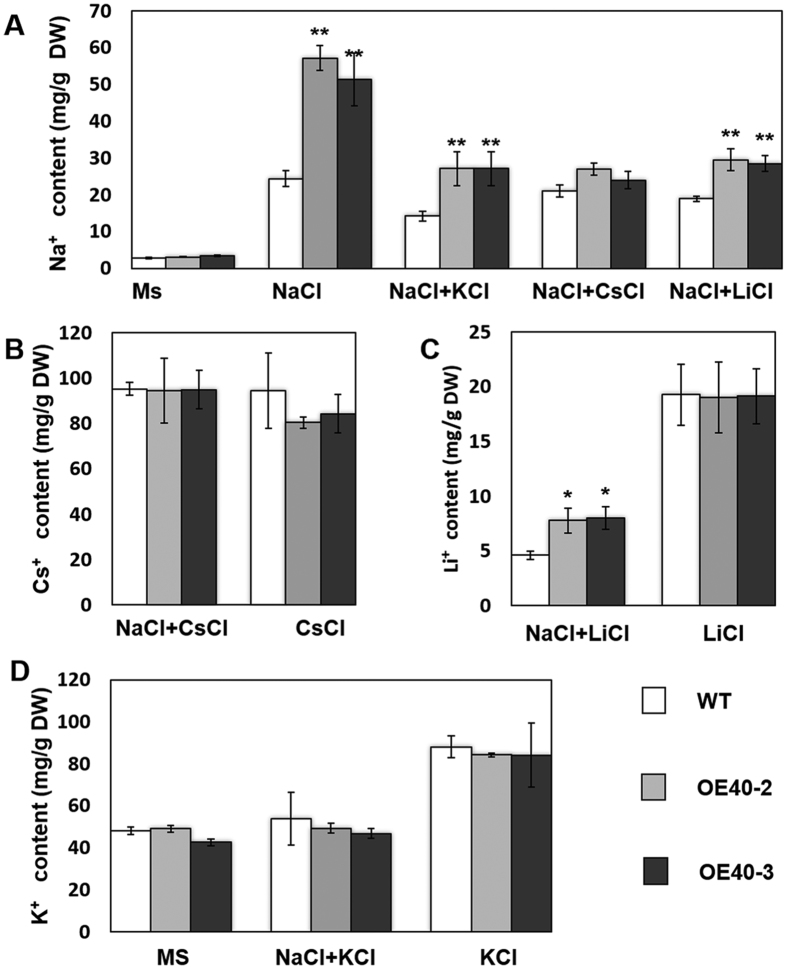
Ion contents in wheat roots treated with NaCl, KCl, LiCl and CsCl. **(A)** Na^+^ contents: the wheat plants were watered with MS, 150 mM NaCl or 75 mM NaCl with 75 mM KCl/CsCl/LiCl. **(B)** Cs contents: the wheat plants were watered with 150 mM CsCl or 75 mM CsCl with 75 mM NaCl. **(C)** Li^+^ contents: the wheat plants were watered with 150 mM LiCl or 75 mM LiCl with 75 mM NaCl. **(D)** K^+^ contents: wheat plants were watered with MS, 150 mM KCl or 75 mM KCl with 75 mM NaCl. The samples were harvested 24 hours later. Bars represent the mean ± SD (n = 3, *P < 0.05, **P < 0.01 by Student’s t-test).

The sodium transport mechanism in plants primarily includes Na^+^ specific channels and nonselective cation channel pathways^1^,^9^,^35^. Therefore, whether the higher Na^+^ content in transgenic lines was related to Na^+^ specific channels or to nonselective cation channels must be determined. In transgenic Arabidopsis and wheat overexpressing *TaCIPK25*, no obvious changes were observed in the K^+^ content between transgenic and control lines under salt treatment (Figs 4C and 5E). For further analysis, the transgenic and wild type wheat were treated with 150 mM KCl for 24 h, and no obvious difference in K^+^ content was observed (Fig. 6D). When seedlings were treated with 75 mM NaCl and 75 mM KCl, transgenic lines accumulated more Na^+^ than that in the wild type, but no significant changes occurred in K^+^ content. Under normal conditions (MS-treated), the Na^+^/K^+^ content in transgenic and wild type seedlings was not different.

In a previous study, the wheat gene *TaLCT1*, a low affinity cation transporter, generated hypersensitivity to Na^+^ in yeast and mediated the uptake of Na^+^, Cs^+^ and Li^+ ^36. In this study, 24 h after treatment with 150 mM CsCl or 150 mM LiCl, the transgenic lines overexpressing *TaCIPK25* did not accumulate more Cs^+^ and Li^+^ than wild-type controls (Fig. 6B,C). However, the addition of 75 mM NaCl with 75 mM CsCl restrained the TaCIPK25-induced Na^+^ accumulation, and Na^+^ did not affect the accumulation of Cs^+^ (Fig. 6B). Additionally, transgenic lines that overexpressed *TaCIPK25* accumulated more Li^+^ only when NaCl was added (Fig. 6C), indicating a likely Na^+^-induced Li^+^ accumulation pathway. In general, based on these results, Na^+^ homeostasis regulated by TaCIPK25 in transgenic wheat lines did not involve nonselective cation channel pathways.

### TaCIPK25 involvement in regulation of Na^+^/H^+^ exchanges

In a previous study using the yeast two-hybrid method, we found that TaCIPK25 interacted with TaCBL1^29^. To further confirm this interaction and determine the localization, TaCBL1 and TaCIPK25 were respectively constructed to the C- and N-terminals of yellow fluorescent protein (YFP) for a bimolecular fluorescence complementation (BiFC) assay and to GFP fusion constructs for intracellular locations. As shown in Supplementary Fig. 6, TaCBL1 was primarily localized at the cell membrane and TaCIPK25 was distributed ubiquitously within the cell. Notably, yellow fluorescent signal was observed at the cell membrane in the BiFC analysis, which indicated the interaction between TaCBL1 and TaCIPK25 in plant cells and a potential functional site.

**Figure 7:**
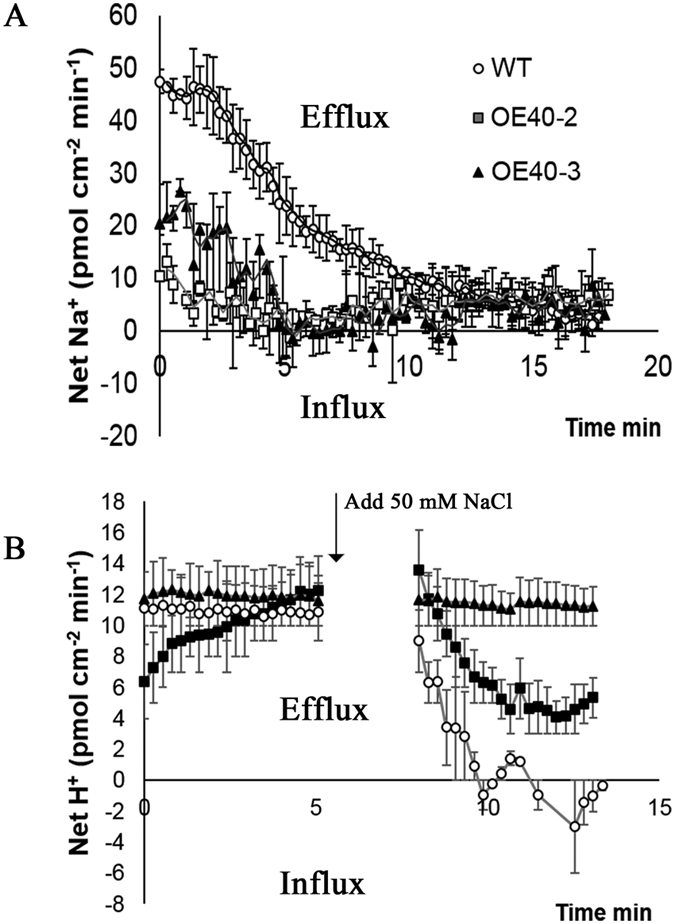
The flux of net H^+^/Na^+^ under salt treatment. **(A)** The net Na^+^ effluxes in roots of wheat plants. The wheat plants were pre-treated with 50 mM NaCl for 3 hours and then transmitted to measuring buffer for non-invasive ion flux measurement by SIET technique. **(B)** The net H^+^ fluxes in roots of wheat plants. The net H^+^ fluxes were measured in measuring buffer added 50 mM NaCl. At least three biological experiments were performed. Bars represent the mean ± SD (n = 3).

To explore whether co-localization of TaCBL1 and TaCIPK25 indicated a potential functional site for ion flux regulation at the cell membrane, we measured the transmembrane ion fluxes noninvasively using the scanning ion-selective electrode technique (SIET). For the measurement of Na^+^ efflux, wheat plants were pretreated with 50 mM NaCl for 3 h and then transferred to measure buffer for Na^+^ flux measurements. As shown in Fig. 7A, the net immediate Na^+^ efflux was lower in transgenic lines than that in control seedlings with NaCl treatment; therefore, overexpression of *TaCIPK25* in wheat likely decreased Na^+^ efflux from cells. For net H^+^ efflux, we found no significant differences between WT and transgenic wheat lines, when wheat plants were put into measuring buffer. However, the H^+^ effluxes of the wild type were significantly more impaired than those of transgenic wheat lines after the addition of 50 mM NaCl (Fig. 7B), which indicated lower H^+^ influxes in transgenic wheat lines. Thus, based on these results, TaCIPK25 was likely involved in regulating Na^+^/H^+^ exchange.

## Discussion

High soil salinity is one of the factors that most restricts plant growth and development, and plants have developed many mechanisms to cope with salt stress. An intriguing question is how this salinity response mechanism is initiated and then transmitted to downstream functional proteins when plants are exposed to a saline environment. ABA is an important signal molecule that plants use to respond to abiotic stress, which also regulates many stress-responsive genes^30^. WRKY transcription factors are both positive and negative regulators of ABA signaling and rapidly activate gene expression in plant defensive processes^31^,^32^. In this study, we found interactions among ABA, WRKY and CIPK in the regulation of ABA pathways. Expression levels of *TaCIPK25* were regulated by ABA and high salinity, and when endogenous ABA was inhibited by fluridone, expression recovered in roots (Fig. 1C), which indicated expression was regulated by an ABA-dependent pathway. Notably, the expression patterns of *TaWRKY9* were in contrast to those of *TaCIPK25* under salinity and ABA application with levels of expression also affected by eliminating endogenous ABA (Fig. 3B). We showed that TaWRKY9 bound directly to the promoter of *TaCIPK25* and negatively regulated the expression levels of *TaCIPK25* (Fig. 2). Therefore, TaWRKY9 likely negatively regulated the expression of *TaCIPK25* through an ABA-dependent pathway under salt stress.

To explore the effects on wheat plants caused by the regulation of *TaCIPK25* expression by TaWRKY9 under salt treatment, transgenic Arabidopsis and wheat plants were generated that overexpressed *TaCIPK25*. In early studies, overexpression of *CIPK*s increased salt tolerance in many plants^25^,^37^,^38^,^39^. In this study, overexpression of *TaCIPK25* in Arabidopsis and wheat had negative effects on salt tolerance with overexpression in transgenic lines leading to the accumulation of more Na^+^ than that in wild-type controls, which indicated a different mechanism for involvement of CIPK in the response to salt stress. At the cellular level, Na^+^ accumulation in plants primarily occurs through two distinct processes, Na^+^ influx and efflux. For Na^+^ uptake, many nonselective cation channels (NSCCs) are identified as Na^+^ permeable channels^40^,^41^,^42^, which also have other cation transportation capabilities. For example, TaLCT1 is a low-affinity cation transporter that mediates the uptake of Na^+^, Li^+^ and Cs^+^, and the uptake of Na^+^ through LCT was inhibited by K^+^ and Cs^+^ in yeast^36^. Our results showed that transgenic wheat lines overexpressing *TaCIPK25* specifically accumulated Na^+^, whereas the transport of Li^+^ and Cs^+^ was unaffected. Moreover, neither Li^+^ nor K^+^ inhibited Na^+^ uptake in these plants (Fig. 6). For Cs^+^, the addition of CsCl eliminated the difference in Na^+^ content between control and transgenic lines (Fig. 6B), implying a potential function related to TaLCT1 or other ion homeostasis mechanism. However, no significant differences in Cs^+^ content between control and transgenic lines were observed under the 150 mM CsCl treatment. Therefore, based on current data, TaCIPK25 was apparently not involved in the regulation of TaLCT1 or other NSCCs. In other studies, the durum wheat SOS1 and Arabidopsis NHX8 (SOS) proteins efficiently mediated not only Na^+^ efflux but also Li^+^ efflux^43^,^44^. Notably, transgenic wheat overexpressing *TaCIPK25* accumulated Li^+^ through a Na^+^-dependent pathway (Fig. 6C), and low levels of Na^+^ effluxes and H^+^ influxes were observed in the *TaCIPK25* overexpressed wheat lines (Fig. 7), which suggested a plausible relationship between TaCIPK25 and SOS pathways with different roles in regulating the Na^+^/H^+^ antiporter. However, based on the current data, we cannot conclude that TaCIPK25 participated in negative regulation of the SOS pathway. Nevertheless, specific Na^+^ transportation was a reasonable explanation for the TaCIPK25-induced salt sensitive phenotype, which also provided a clue for the exploration of potential target proteins of TaCIPK25 in future work.

**Figure 8:**
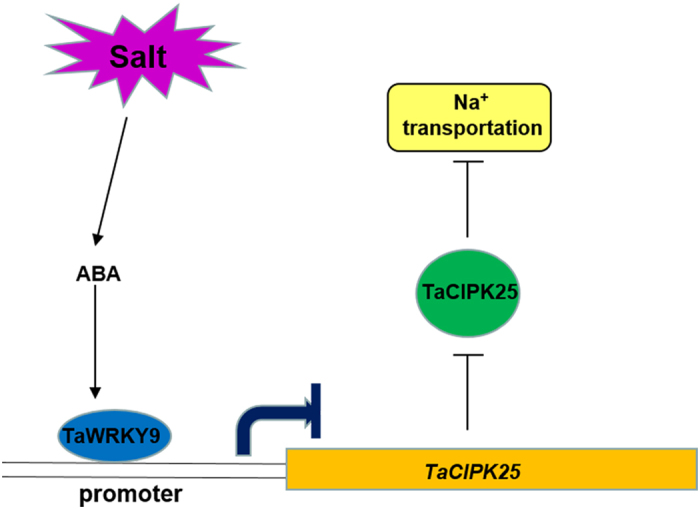
Hypothetical model of TaCIPK25 function. Salt stress signals were perceived by ABA signaling molecule and *TaWRKY9* were up-regulated to inhibit the expression of *TaCIPK25* roots and further affect Na^+^ transportation.

In conclusion, this study provides insights into the mechanisms of gene expression regulation and salt response of TaCIPK25 in wheat (Fig. 8). Under nonsaline conditions, *TaCIPK25* is persistently expressed in wheat roots and might be involved in uptake of nutritional Na^+^. When plants encounter high-salinity stress, ABA, as an important signal molecule, regulates expression of many stress-responsive genes, including *TaWRKY9*. The ABA-dependent pathway to induce the transcription factor gene *TaWRKY9* which represses *TaCIPK25* expression in wheat roots rapidly induced and further affected Na^+^ transportation. The sensitive phenotypic characteristics that were observed in transgenic wheat were closely related to the expression levels of *TaCIPK25,* which did not decline and even maintained a higher level of expression than that of wild-type wheat controls under the high salinity treatment. Therefore, the down-regulation of *TaCIPK25* mediated by TaWRKY9 is a protection mechanism to cope with high salinity environments in wheat, which also provides useful information for the genetic manipulation of wheat to improve salt tolerance. Studies are also underway to examine specific Na^+^ channels that directly interact with TaCIPK25 protein with the aim to better understand the involvement of TaCIPK25 in the salt response pathway.

## Methods

### Wheat treatments and qRT-PCR

The wheat cultivar Chinese spring was used in this study. For expression of *TaCIPK25* in wheat tissues, seedling stage tissues (coleoptiles, roots, stems and leaves) were obtained from sterile seedlings, and flowering stage tissues (roots, stems, leaves, flag leaves, pollen and pistils), embryos (approximately 15 d after anthesis) and endosperm (approximately 15 d after anthesis) were cut from wheat plants grown in soil. For stress response analyses, 7-day-old wheat seedlings were treated with ABA (100 μM), H_2_O_2_ (10 μM) and NaCl (200 mM). For treatments with inhibitors, seedlings were first pretreated with fluridone (10 μM) for 2 h and then exposed to H_2_O_2_ (10 μM) + fluridone (10 μM) and NaCl (200 mM) + fluridone (10 μM). All samples were subsequently frozen in liquid nitrogen and stored at −80 °C for extraction of total RNA.

Total RNA was extracted with a Plant Total RNA kit (Zoman Biotechnology, Beijing, China) and used to synthesize first-strand cDNA using a FastQuant RT Kit (Tiangen, Beijing, China). SuperReal PreMix kits (Tiangen, Beijing, China) were used for qRT-PCR. The *actin* gene was used as the housekeeping control gene. Transcript level of a gene relative to the housekeeping control gene was the value of expression. All primers for gene expression assays are listed in Supplementary Table 1. Each expression analysis was performed independently at least four times.

### Assay to determine binding of WRKYs to *cis*-elements of *TaCIPK25* promoter region

To analyze putative *cis*-elements in the promoters of *TaCIPK25* gene, a 1,000 bp upstream region of the gene was extracted from wheat genomic sequences and was searched for a *cis*-elements using PLACE (http://www.dna.affrc.go.jp/PLACE/signalup.html). The putative W-boxes (W1-W5, each containing three tandem repeat copies) were subcloned to PHis2.0 vectors, and WRKYs were constructed into pGADT7 vectors. The detailed experimental procedure followed that in a previous publication by Wang *et al*.^34^. DPI-ELISA assays followed the description in a previous publication^45^. Recombinant proteins were obtained by a prokaryotic expression system (expressed in *E. coli* BL21). The biotinylated complementary oligonucleotides were ordered from a company (BGI.tech, China). The relative GUS activity assays were performed, primarily, as described elsewhere^46^,^47^. All tested tobacco plants (*TaWRKY1, TaWRKY9* and *TaWRKY44*) were transformed by constructs (*proTaCIPK25:GUS, 35S:LUC*) using the *Agrobacterium*–mediated transient expression procedure. The *35S:LUC* construct was used as a transformation efficiency control. The level of expression (relative to the housekeeping gene) was similar in all transgenic tobacco lines used in this study.

### Plant transformations

To generate transgenic Arabidopsis overexpressing *TaCIPK25*, the CDS of *TaCIPK25* was constructed to pBI121 vector driven by cauliflower mosaic virus 35S (*CaMV* 35S) promoter, and pBI121-TaCIPK25 was transferred into *Agrobacterium tumefaciens* strain LBA4404. The transformation of Arabidopsis was performed with the floral dip method^48^,^49^. T_0_ positive seeds were selected on MS medium containing kanamycin (50 mg L^−1^), and T_2_ generation seeds were selected for further analysis.

The wheat transformation protocol was conducted based on a particle bombardment method^50^. Genomic DNA extracted from leaf tissue by a PlantGen DNA Kit (Beijing ComWin Biotech, Beijing, China) was used for T_0_ positive transgenic lines with specific primers with PCR (Supplementary Table 1). PCR products were confirmed by sequencing.

### Assay of stress tolerance phenotype in transgenic lines and control plants

Seeds of WT, VC and transgenic lines were germinated on MS medium after surface sterilization. Four-leaf stage seedlings (10 d) were transferred to MS medium containing 150 mM NaCl for salt stress analysis. For salt assays in soil, 10-day-old seedlings were irrigated with 200 mM NaCl at 5-day intervals. For anti-oxidation experiments in soil, 10-day-old seedlings were uniformly sprayed with 10 μM methyl viologen at 5-day intervals.

After germination of wheat seeds, wheat seedlings were grown in water for 10 d. For salt tolerance assays, 10-day-old wheat seedlings were transferred to MS liquid medium with 150 mM NaCl with the medium refreshed daily.

### Measurements of Na^+^/H^+^ fluxes, ions and H_2_O_2_ content

The scanning ion-selective electrode technique (SIET, Equshine Co. Ltd., Shanghai, China; Applicable Electronics, LLC, New Haven, CT, USA) was used to measure Na^+^ and H^+^ fluxes at the elongation zone of wheat seedling roots in measuring buffer (0.1 mM KCl, 0.5 mM NaCl, 0.2 mM Na_2_SO_4_, 0.1 mM MgCl_2_, 0.1 mM CaCl_2_, 0.3 mM MES, pH 6.0). The procedure used to measure ion fluxes was described in previous reports^51^,^52^. Briefly, electrodes were made from glass micropipettes and filled with selective liquid ion-exchange cocktails (Na: 100 mM NaCl; H: 40 mM KH_2_PO_4_; and 15 mM NaCl, pH 7.0) for Net Na^+^/H^+^ measurements. Only electrodes with slopes >50 mV/decade were used in this study. Wheat seedlings were placed in measuring buffer without or with 50 mM NaCl for H^+^ flux measurements. For net Na^+^, wheat seedlings pretreated with 50 mM NaCl for 3 h were transferred to measuring buffer (without NaCl). The fluxes of target ions were determined by ion concentration gradients between two positions close to the plant material in a preset excursion (30 μm). Further details on data processing and calibration of SIET are found on the following web site: http://marlin.bio.umass.edu/biology/kunkel/nvp_umass.html.

For Arabidopsis, the leaves of seedlings treated with 150 mM NaCl were harvested at 10 d for ion and H_2_O_2_ measurements. For ion assays in wheat plants, 10-day-old seedlings were transferred to MS liquid medium with or without ions (Na^+^, K^+^, Li^+^ and Cs^+^) and grown for 24 h. To measure the ion content, all samples were dried at 75 °C for 3 d, weighed and extracted with 1 N HNO_3_+15% H_2_O_2_ at 121 °Cfor 30 min. Ion concentrations were determined with an atomic absorption spectrophotometer (Analytik, Jena, Germany). The H_2_O_2_ content was assessed using a commercially available kit (Nanjing Jiancheng Bioengineering Institute, Nanjing, China).

### Subcellular localizations and biomolecular fluorescence complementation (BiFC)

The cDNA of *TaCBL1* and *TaCIPK25* was subcloned into modified pBI121 vectors (fused with GFP) and BiFC vectors (YNE-TaCBL1 and YCE-TaCIPK25) using specific primers (Supplementary Table S1) for transient expression in onion epidermal cells. These vectors and PM-RK (a plasma membrane-localized marker based on the full-length coding region of *AtPIP2A*) were transiently expressed in onion epidermal cells using a Biolistic Particle Delivery System (PDS-1000/He; Bio-Rad, CA, USA)^53^. Fluorescence was observed by inverted fluorescence microscopy (IX71; Olympus, Tokyo, Japan) after incubation at 25 °Cfor 16–24 h on MS medium.

## Additional Information

**How to cite this article**: Jin, X. *et al*. Wheat CBL-interacting protein kinase 25 negatively regulates salt tolerance in transgenic wheat. *Sci. Rep.* **6**, 28884; doi: 10.1038/srep28884 (2016).

## Supplementary Material

Supplementary Information
