# Supplementary material for: Wheat CBL-interacting protein kinase 25 negatively regulates salt tolerance in transgenic wheat
Source: Sci Rep. 2016 Jun 30;6:28884. doi: 10.1038/srep28884 (PMC4928124; doi:10.1038/srep28884)
Supplement: Supplementary Information [file srep28884-s1.doc]

**Wheat** **CBL-interacting protein kinase 25 negatively regulates salt tolerance in transgenic wheat**

Xia Jin^†^, Tao Sun^†^, Xiatian Wang, Peipei Su, Jingfei Ma, Guangyuan He*, Guangxiao Yang*

† Equal contributors

*Corresponding authors

The Genetic Engineering International Cooperation Base of Chinese Ministry of Science and Technology, The Key Laboratory of Molecular Biophysics of Chinese Ministry of Education, College of Life Science and Technology, Huazhong University of Science & Technology, Wuhan 430074, China

**Supplementary Figure**


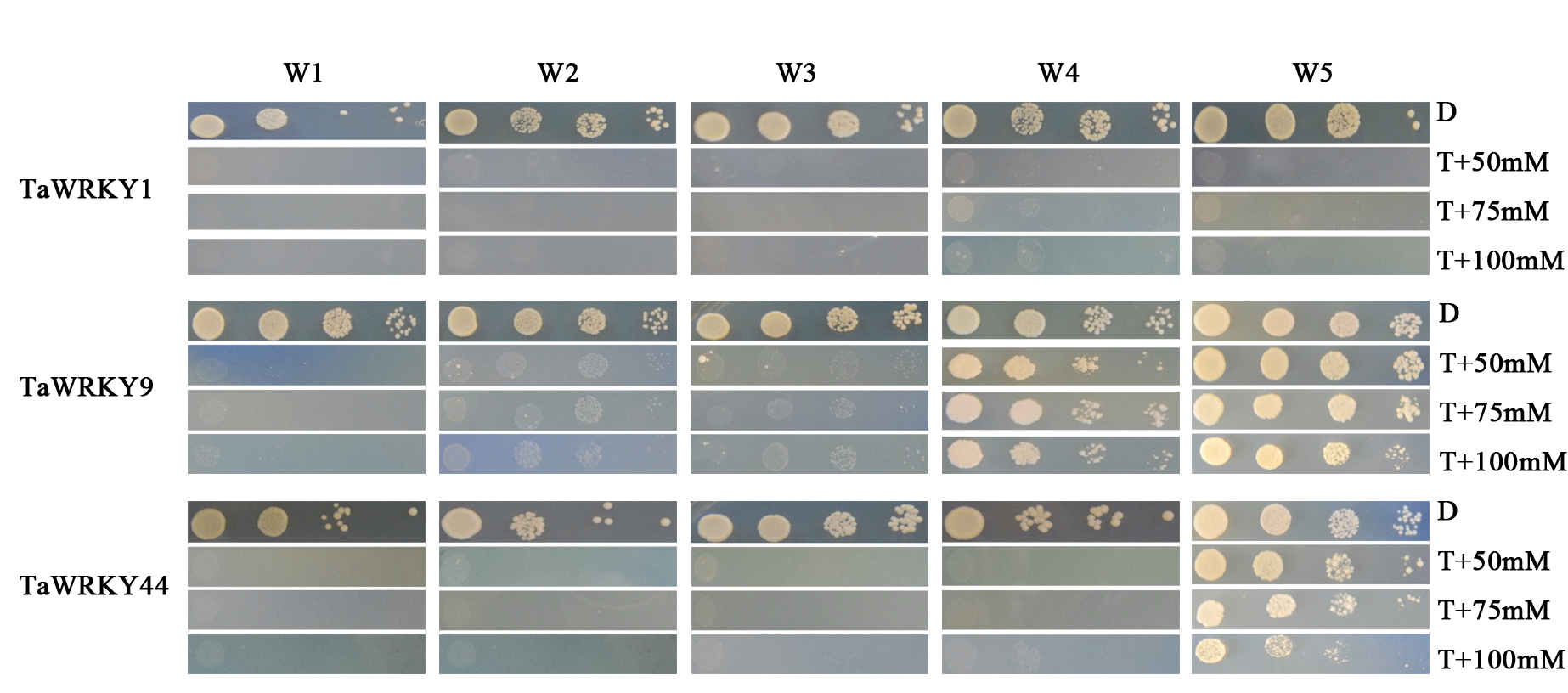


**Supplementary Figure 1**. Binding activity analysis between W-box on TaCIPK25 and other three TaWRKYs (TaWRKY1, TaWRKY9 and TaWRKY44) promoter regions and using yeast one-hybrid system. The coding sequences of three TaWRKYs were constructed to PGADT7 vector as the effector constructs. The effector and reporter constructs were co-transformed into the yeast strain Y187. Positive transformants were selected on SD/-His/-Leu/-Trp (TDO) medium with different concentrations of 3-AT (50 mM, 75 mM and 100 mM).


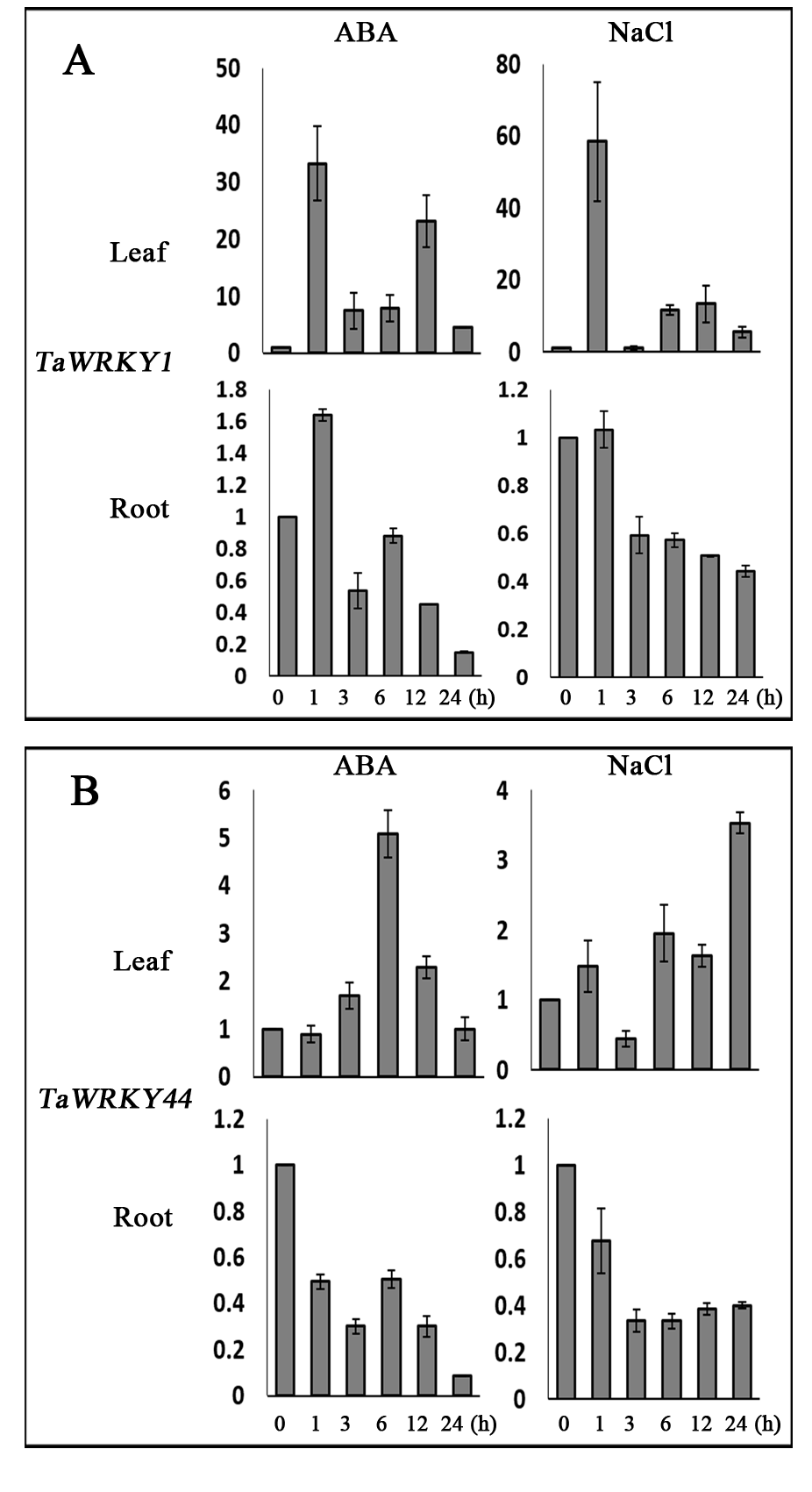


**Supplementary Figure 2**. Expression patterns of *TaWRKY1* and *TaWRKY44* in wheat plants treated with ABA (10 μM) and NaCl (200 mM), respectively. The Y-axis represents the relative expression levels compared with controls (0 h). Three biological experiments were performed.


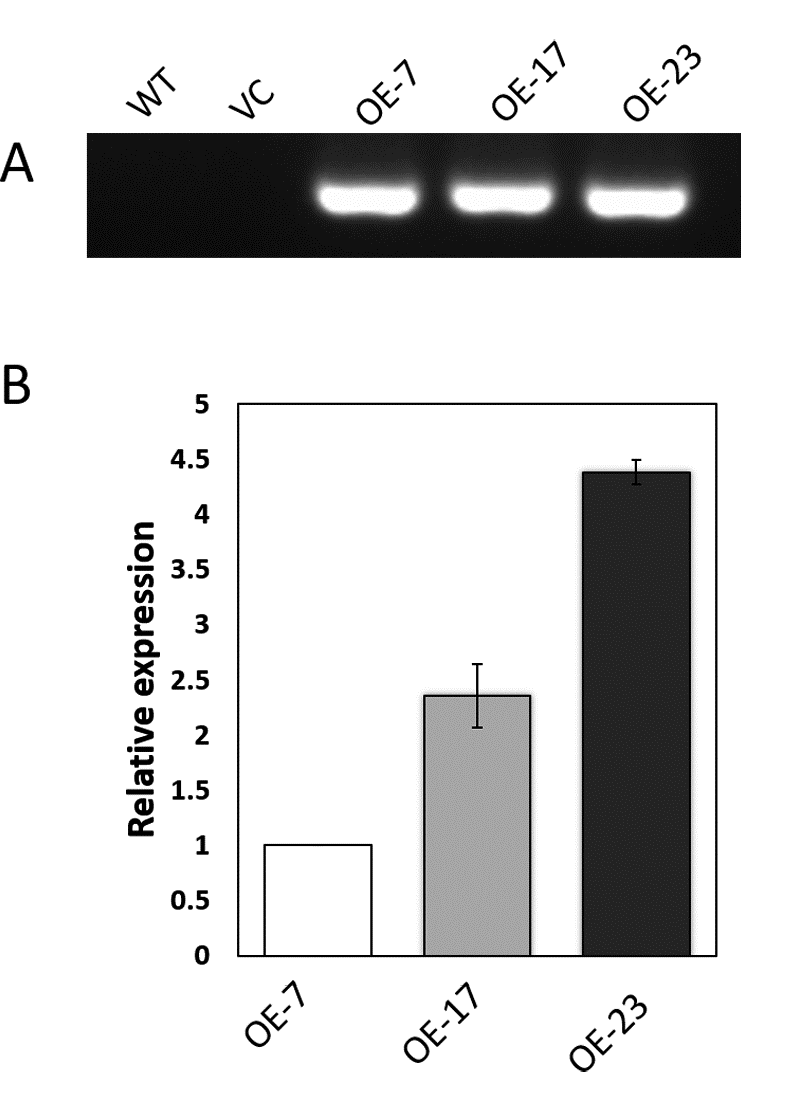


**Supplementary Figure 3**. The expression analyses of *TaCIPK25* in transgenic Arabidopsis. A, detection of expression of *TaCIPK25* in transgenic and control Arabidopsis lines by RT-PCR method. B, The relative expression levels of *TaCIPK25* in three assayed transgenic lines. Three biological experiments were performed.


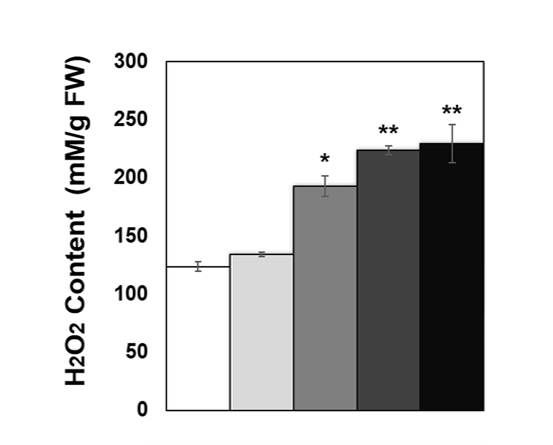


**Supplementary Figure 4**. The H_2_O_2_ contents of Arabidopsis after 10-day salt treatment. Each value is the mean (±SD) of six measurements.


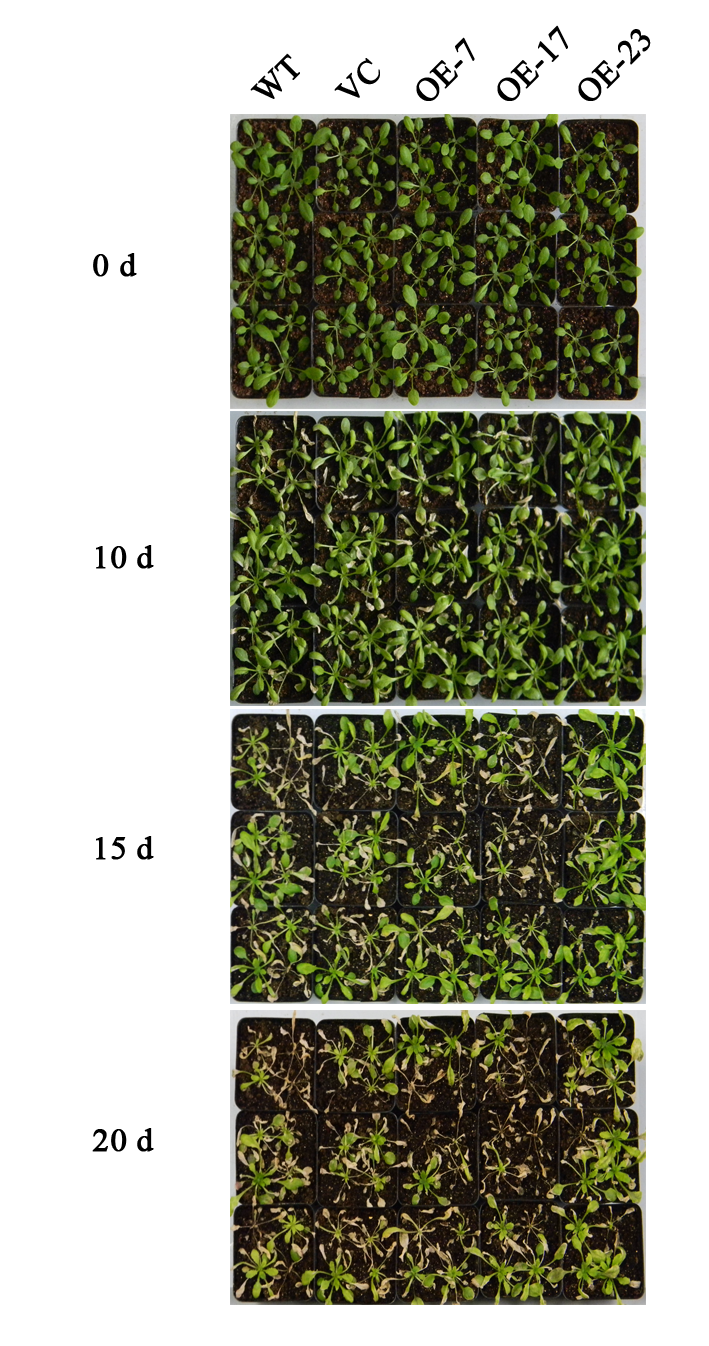


**Supplementary Figure 5**. Anti-oxidation experiment in transgenic Arabidopsis treated with methyl viologen. Ten-day-old seedlings were sprayed with 10 μM methyl viologen at 5-days interval and the photos were took at 10, 15 and 20 days after salt treatment.


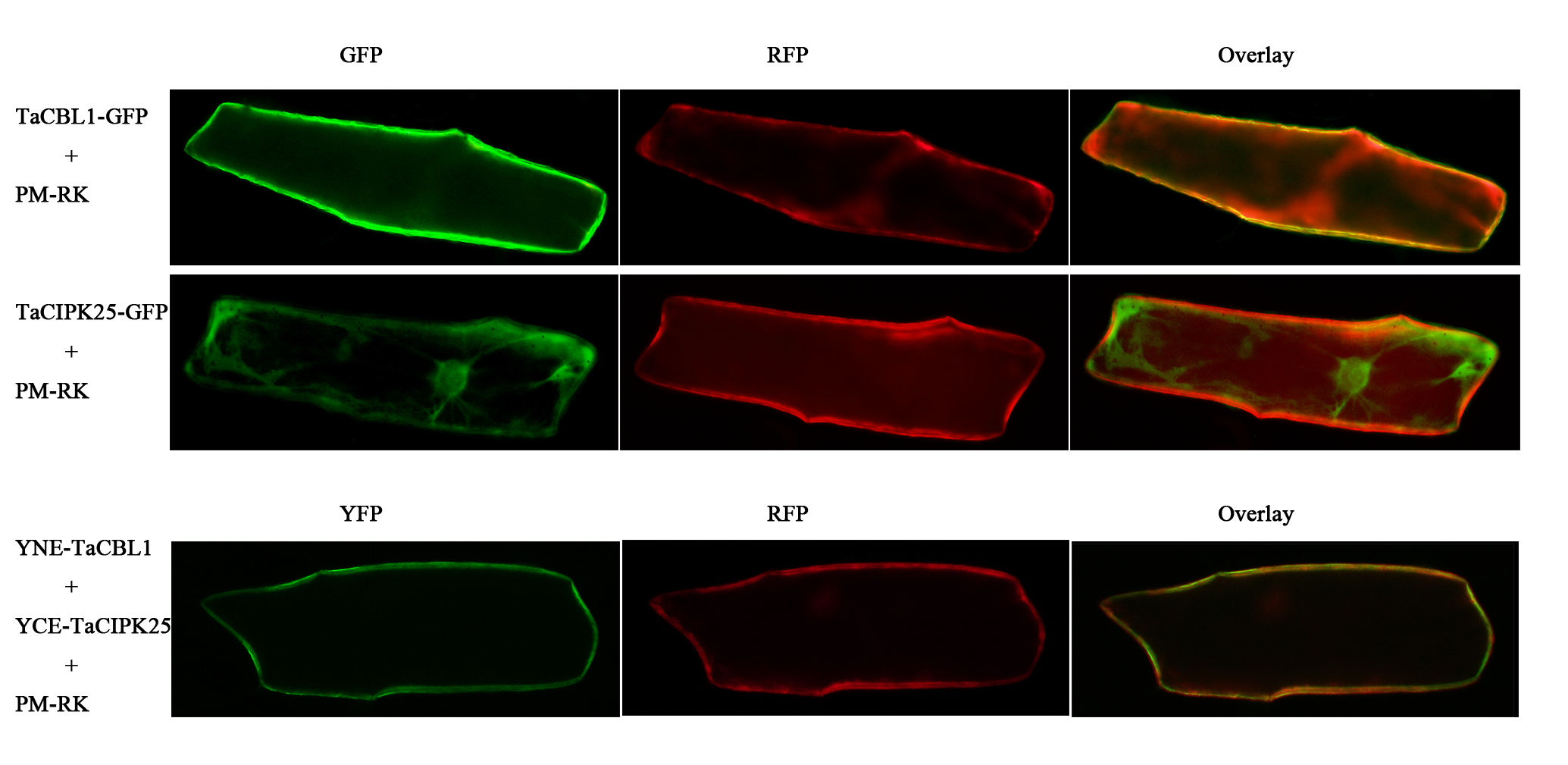


**Supplementary Figure 6**. Localizations and BiFC analyses of TaCBL1/TaCIPK25 in onion epidermal cells. The cDNA of *TaCBL1* and *TaCIPK25* were sub-cloned into modified pBI121 vectors (fused with GFP) and BiFC vectors (YNE-TaCBL1 and YCE-TaCIPK25). The PM-RK was used as the plasma membrane localized maker. Three biological experiments were performed, which produced similar results.

**Supplementary Table 1** Forward and reverse primers used in gene expression analyses and vector constructions

| Names | Forward primers | Reverse primers | **Assays** |
| --- | --- | --- | --- |
| *TaCIPK25* | gcctgatctctgtgactctg | gaaaccgaggtgtgcatgcc | Genes expression analyses |
| *TaWRKY1* | cgtccttcttctcgtcggtga | tcttccttctcttggagcagtgg |
| *TaWRKY9* | gtccaacgccacggtcaa | tgctgctgctgctcttgtt |
| *TaWRKY44* | ccaacggcggtgataactacat | gctactggatgctgccttctg |
| *TaActin* | cttgtatgccagcggtcgaaca | ctcataatcaagggccacgta |  |
| *AtUbi* | cggaaagcagttggaggatgg | cggagcctgagaacaagatgaag |  |
| *TaWRKY1* | GAATTCatggccgtggacctcatggg | CTGCAGctcagacgtgcccgttgccg | Y1H assays |
| *TaWRKY9* | CATATGatgtcctcctccacggggag | GGATCCctagcagaggagcgactcg |
| *TaWRKY44* | gAATTCatgtcccatcagcaagcgtt | CTgCAgttatgttatctcattttcttct |
| *TaCIPK25* | AAGCTTcaagcaaagaacaacaac | CCCGGGcgccgtcgccgcttc | Gus relative activity assays in tobacco |
| SP | cgcgggctgagtaaaggagaagaact | gagcacttatttgtatagttcatcca | Detection of exogenous *TaCIPK25* in overexpression wheat |
| TaCBL1 | GGATCCatggggtgcatccagtc | CCCGGGtgtaacgatatcatcaac | Subcellular localization |
| TaCIPK25 | GGATCCatgggggatcggccaaag | CCCGGGcgcaacattcaacgtcg |
| TaCBL1 | GGATCCatggggtgcatccagtc | CCCGGGtgtaacgatatcatcaac | BiFC |
| TaCIPK25 | GGATCCatgggggatcggccaaag | CCCGGGcgcaacattcaacgtcg |
